# A study of noninvasive fractional flow reserve derived from a simplified method based on coronary computed tomography angiography in suspected coronary artery disease

**DOI:** 10.1186/s12938-017-0330-2

**Published:** 2017-04-14

**Authors:** Changzheng Shi, Dong Zhang, Kunlin Cao, Tao Zhang, Liangping Luo, Xin Liu, Heye Zhang

**Affiliations:** 1 Medical Imaging Center, The First Affiliated Hospital, Jinan University, 613 Huangpu W Ave, Tianhe District, Guangzhou, 510630 Guangdong Province China; 2 Research and Development Department, Shenzhen Keya Medical Technology, Co., Ltd., Longgang District, Shenzhen, 518116 Guangdong Province China; 3 Shenzhen Institutes of Advanced Technology, Chinese Academy of Sciences, 1068 Xueyuan Ave. Xili University Town, Nanshan District, Shenzhen, 518055 Guangdong Province China

**Keywords:** CCTA, FFR_CTA_, FFR, CFD

## Abstract

**Background:**

The invasive fractional flow reserve has been considered the gold standard for identifying ischaemia-related stenosis in patients with suspected coronary artery disease. Determining non-invasive FFR based on coronary computed tomographic angiography datasets using computational fluid dynamics tends to be a demanding process. Therefore, the diagnostic performance of a simplified method for the calculation of FFR_CTA_ requires further evaluation.

**Objectives:**

The aim of this study was to investigate the diagnostic performance of FFR_CTA_ calculated based on a simplified method by referring to the invasive FFR in patient-specific coronary arteries and clinical decision-making.

**Methods:**

Twenty-nine subjects included in this study underwent CCTA before undergoing clinically indicated invasive coronary angiography for suspected coronary artery disease. Pulsatile flow simulation and a novel boundary condition were used to obtain FFR_CTA_ based on the CCTA datasets. The Pearson correlation, Bland–Altman plots and the diagnostic performance of FFR_CTA_ and CCTA stenosis were analyzed by comparison to the invasive FFR reference standard. Ischaemia was defined as an FFR or FFR_CTA_ ≤0.80, and anatomically obstructive CAD was defined as a CCTA stenosis >50%.

**Results:**

FFR_CTA_ and invasive FFR were well correlated (r = 0.742, P = 0.001). Slight systematic underestimation was found in FFR_CTA_ (mean difference 0.03, standard deviation 0.05, P = 0.001). The area under the receiver-operating characteristic curve was 0.93 for FFR_CTA_ and 0.75 for CCTA on a per-vessel basis. Per-patient accuracy, sensitivity and specificity were 79.3, 93.7 and 61.5%, respectively, for FFR_CTA_ and 62.1, 87.5 and 30.7%, respectively, for CCTA. Per-vessel accuracy, sensitivity and specificity were 80.6, 94.1 and 68.4%, respectively, for FFR_CTA_ and 61.6, 88.2 and 36.8%, respectively, for CCTA.

**Conclusions:**

FFR_CTA_ derived from pulsatile simulation with a simplified novel boundary condition was in good agreement with invasive FFR and showed better diagnostic performance compared to CCTA, suggesting that the simplified method has the potential to be an alternative and accurate way to assess the haemodynamic characteristics for coronary stenosis.

## Background

Coronary artery disease (CAD), the most common type of heart disease, has become the leading cause of death among Chinese adults [1]. The presence of myocardial ischaemia is the most important risk factor for an adverse outcome, and the revascularization of ischaemia-related stenotic coronary lesions can improve patients’ functional status in the clinic [2]. Coronary revascularization is often performed based on semi-quantitative measures of stenosis during invasive coronary angiography (ICA) [3]. However, the relationship between coronary stenosis severity and myocardial ischaemia is unreliable. In lesions with stenosis <50, 50–70% and >70%, only 9, 18 and 57% of lesions are ischaemia causing [4]. This suggests that basing clinical treatment decisions on stenosis severity alone would result in unnecessary procedures; physiological information may be more important.

At present, the fractional flow reserve (FFR) has been regarded as the gold standard in the assessment of haemodynamic characteristics for coronary stenosis [5], and it is recommended for clinical treatment decision making before coronary revascularization [6]. In the FAME (fractional flow reserve versus angiography for multivessel evaluation) trial, FFR-guided revascularization (revascularization for lesions with FFR ≤0.80) led to an approximately 28% lower rate of major adverse cardiac events compared with an angiography-guided strategy [7]. However, FFR is applied to guiding management in less than 10% of percutaneous coronary intervention (PCI) cases [8]. The high cost of the coronary pressure wire and the invasive medical operation may hinder the application of FFR measurements in the clinic [9, 10].

Coronary computed tomographic angiography (CCTA) has emerged as a non-invasive method to visualize CAD and assess anatomic stenosis severity [11–13]. In recent years, the advances in computational fluid dynamics (CFD) have made it possible to simulate and calculate the coronary flow and pressure from anatomic imaging data [14]. Based on the reconstructions from CCTA images, FFR without additional medications could be calculated. Integrating anatomic and functional information, non-invasive FFR derived from CTA (FFR_CTA_) may be an available and cost-effective method to identify individuals who will or will not benefit from coronary revascularization.

Several randomized trials have shown that the performance of FFR_CTA_ was superior to CTA stenosis for diagnosing ischaemic lesions [15–17]. However, the time span to simulate the transient CFD and calculate FFR_CTA_ is usually 6 h [15] or 1–4 h [17] per examination. Using a reduced-order algorithm, Coenen reported on-site computational FFR_CTA_ software requiring only 5–10 min to calculate the CFD per patient [18]. However, this method only had a moderate to good correlation (r = 0.59). Recently, Zhang et al. employed steady state flow simulation to obtain FFR_SS_ and reduced the computational time to 0.5–2 h, together with a good correlation between FFR_SS_ and invasive FFR (r = 0.843) [19]. In addition to the steady state flow simulation applied in Zhang’s research, we evaluated the effect of pulsatile flow on FFR_CTA_ based on a simplified calculation method for the outflow boundary parameters. The aim of this study was to investigate the correlation between the simplified FFR_CTA_ and invasive FFR in patient-specific coronary arteries, together with the diagnostic performance of the simplified FFR_CTA_ in the clinic, and to discuss the feasibility of using this simplified method in identifying ischaemia-related stenosis of CAD.

## Methods

### Populations

This study was approved by the ethical review committee of the First Affiliated Hospital of Jinan University (Guangzhou, Guangdong, China). Since this study is a retrospective study, the informed consent was waived and anonymized data were used for analysis. Coronary CTA performed less than 60 days before scheduled non-emergent ICA and FFR measurement was required for inclusion. Exclusion criteria included individuals who were unable to provide informed consent; complete occlusion of the coronary arteries; significant arrhythmia; non-cardiac illness with life expectancy <2 years; pregnant state; previous coronary intervention or coronary bypass surgery; allergy to iodinated contrast; contraindications to beta-blocking agents, nitroglycerin, or adenosine; and suspected acute coronary syndrome. Eventually, 29 patients in total were included in this study. The average age ranged from 54 to 82 years old (68.1 years old ± 8.4 years), and the patients were diagnosed with cardiovascular disease between March 15, 2013 and June 23, 2015.

### Coronary CTA acquisition and analysis

Coronary CTA was performed using an MDCT volumetric scanner with 320-detector rows (Aquilion ONE, Toshiba, Otawara, Japan). All the procedures followed the Society of Cardiovascular Computed Tomography guidelines [20]. Oral beta-blockers were administered, targeting a heart rate of <60 beats/min. CCTA data were obtained at both systole and diastole. Experienced radiologists evaluated luminal diameter stenosis in each coronary artery segment using an 18-segment coronary model before ICA [21]. Significant obstruction was defined as luminal stenosis >50% in the main coronary arteries.

### ICA and FFR measurement

ICA was performed according to a standard protocol when the severity of stenosis in a major coronary artery was quantified as more than 50% [22]. Invasive FFR was performed to obtain physiology measurements for clinical indications in significant stenosis. According to the protocol, an FFR pressure wire (PressureWire Aeris/Certus, St. Jude Medical, St. Paul, USA) was positioned distal to the stenosis of interest, at least 3 cm downstream of the lesion, and then hyperaemia was induced by intravenous infusion of adenosine at 140 μg/kg/min [23]. FFR was calculated by dividing the mean distal coronary pressure (mPd) by the mean aortic pressure (mPa) during hyperaemia. The FFR was considered diagnostic of ischaemia at a threshold of 0.80 or less [24].

### Model establishment

**Fig. 1:**
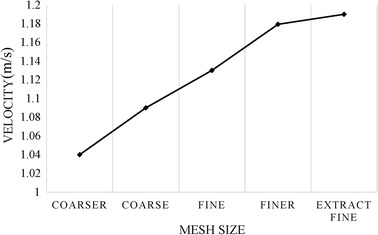
Mesh independent test for the mesh generation procedure. Five densities of meshes were generated for one geometry (coarser, coarse, fine, finer and extra fine) and simulations were performed. The maximum velocity values at the center of the aortic ostium under each density of mesh was recorded for the evaluation of convergence. The test showed that convergence was reached at finer mesh

Patient-specific coronary arterial geometries were reconstructed from 29 sets of CTA image data. By dividing the cross-sectional area of the stenosis by the normal segment proximal to the lesion, 36 lesions were identified as a stenosis by anatomic evaluation. Details of the coronary geometries were determined by the distribution of the contrast agent. Because the coronary lumen was compressed during systole and was unable to be distinguished from the surrounded tissue, the diastole data were used for geometric reconstruction. Vessels were reconstructed offline using Mimics, commercial 3-D reconstruction software (Materialise NV, Leuven, Belgium). The mesh of the geometries was generated using a non-structural mesh with tetrahedron elements. The mesh independence test was performed such that different densities of the meshes were generated in one model. The mesh sizes ranged from coarse (approximately 17,100 nodes with 85,600 elements) to fine (approximately 32,800 nodes with 545,820 elements) such that five mesh sizes were generated in total, as shown in Fig. 1. CFD simulation was performed using each mesh, and the maximum velocities from the calculation were considered indexes from which the values were obtained at the same point of the geometry (the centre of the aortic ostium). Convergence of the test was obtained when the difference of the values between two mesh densities was less than 0.1%. The test results indicated that the standard of the finer mesh approach was appropriate for simulations.

### CFD configuration and FFR_CTA_ computation

Focusing on the haemodynamics in the coronary artery at the peak flow velocity phase, the flow distribution was assumed to be fully developed in this study. Assumptions were made regarding the simulations that the blood flow was incompressible, laminar and Newtonian; the blood viscosity and density were constant at 0.0035 Pa s and 1056 kg/m^3^, respectively [25].

The momentum and mass conservation of flow was solved using Navier–Stokes governing equations as follows:

1 $$ \uprho \left( {\frac{\text{du}}{\text{dt}} + {\text{u}} \cdot \nabla {\text{u}}} \right) = - \nabla p + \upmu \nabla^{2} {\text{u}} + f, $$

2 $$ - \nabla \cdot {\text{u }} = \, 0, $$

where ρ is the density of blood, u is the velocity field, p is the pressure, μ is the viscosity, and f is the body force per unit volume. All data were obtained while the patients were at rest, and because an external force was not involved, f was assumed to be zero [26].

Because pulsatile flow simulation was applied in the present study, the lumped parameter model was implemented for the outflow boundaries. The lumped parameter model (LPM) consisted of resistances and compliances. To achieve the physiological flow condition in arteries, patient-specific parameter values were calculated according to the literature [19, 27]. In brief, the mean flow rate to the coronary arteries was calculated based on the average physiological condition that the flow to the coronary arteries consumed 4% of the stroke volume and the ratio of the blood flow between the left and right coronary arteries was 7 to 3 [28]; the relationship between the resistance of each outlet and the total flow in the coronary arteries was determined by the scale of the branch and the mean inlet pressure/flow rate [19]. Then, the resistances of the LPM of each outlet were calculated according to the relationship of the resistances between normal upstream and downstream. The walls of the vessels were assumed to be rigid and to have no-slip boundaries. The normal flow rate of the aorta ostium was implemented at the inflow boundary [27]. For comparison of the accuracy and the effectiveness, the steady state method [19] was also implemented to calculate FFR_SS_ in the present study.

**Fig. 2:**
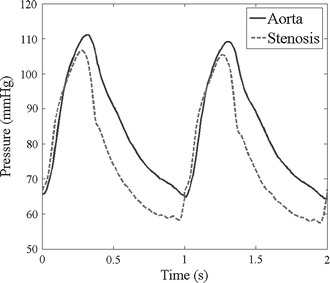
The pressure waveform at the aorta and the stenosis from the transient simulation. The pressure dropped due to the stenosis compared to the pressure of the aorta. The FFR_CTA_ was calculated as the ratio dividing the average pressure at the stenosis in one period of heart cycle by the average pressure at the ostium of the coronary artery in the aorta. The FFR_CTA_ value presented in the figure was, for example, 0.88

Simulations were carried out using COMSOL Multiphysics (COMSOL AB, Stockholm, Sweden), and a multifrontal massively parallel sparse direct solver (MUMPS) was applied to the simulations. FFR_CTA_ was calculated by dividing the average pressure at the stenosis by that at the ostium of the coronary artery. The pressure waveform was extracted from the simulations (e.g., Fig. 2), and FFR_CTA_ was calculated over one heart cycle period, similar to the measurement procedure during clinical practice. The FFR_CTA_ based on the simplified method was calculated under the same condition of the computational platform, and the values were extracted directly from the calculations.

### Statistical analysis

Pearson correlation and Bland–Altman plots were performed to investigate the relationships between FFR_CTA_ and invasive FFR on a per-vessel basis. Invasive FFR was used as the gold standard (FFR ≤ 0.8) to assess the diagnostic performance of FFR_CTA_ and the luminal diameter stenosis. A patient was considered positive if any vessel had FFR ≤0.8, and the vessel with the most adverse clinical status was selected to represent a given patient (minimum FFR, minimum FFR_CTA_ and maximum CCTA stenosis). FFR_CTA_ ≤0.8 was used as the threshold to identify the ischaemic lesions in this study, as well as stenosis >50%. Diagnostic performance on a per-patient and -vessel basis was analyzed, including accuracy, sensitivity, specificity, positive predictive value (PPV), negative predictive value (NPV), positive likelihood ratio (+LR), and negative likelihood ratio (−LR). The area under the receiver-operator characteristics curve (AUC) was also measured for CCTA stenosis and FFR_CTA_. The AUCs were compared by the DeLong method. A P value less than 0.05 was deemed to be statistically significant. All the analyses were performed on SPSS (version 14, Chicago, IL, USA) and MedCalc Software (MedCalc, Mariakerke, Belgium).

## Results

### Baseline characteristics

The study population included 29 patients who underwent coronary CTA and ICA. Baseline characteristics are listed in Table 1. The mean interval between the coronary CTA and FFR was 4.3 days (range 0–14 days), with no adverse events or revascularization between the tests. FFR and FFR_CTA_ were evaluated in a total of 36 coronary vessels. The patient and vessel characteristics according to coronary CTA, FFR_CTA_, FFR_SS_ and FFR are presented in Table 2. On a per-vessel basis, the mean values of measured FFR, FFR_CTA_ and FFR_SS_ were 0.81 ± 0.07, 0.78 ± 0.08 and 0.78 ± 0.07, respectively, and the luminal diameter stenosis evaluated from the CCTA images was 68 ± 15%. Of the 36 vessels, significant obstruction was observed in 75% of the vessels, and 47.2% of the vessels had functionally significant stenosis with FFR ≤0.8.

**Table 1:** Baseline characteristics

Characteristics	Mean ± SD/n (%)
Age, years	68.1 ± 8.4
Male	16 (55.2%)
Diabeta	12 (41.4%)
Hypertension	21 (72.4%)
Hyperlipidemia	19 (65.5%)
Body mass index, kg/m^2^	25 ± 4
Current smoker	13 (44.8%)
Cardiovascular history	
Previous myocardial infarction	1 (3.4%)
Previous PCI	0
Previous CABG	0
Left ventricular ejection fraction, %	60 ± 7
Medications	
Aspirin	15 (51.7%)
Beta-blocker	23 (79.3%)
Nitrate	19 (65.5%)
Statins	17 (58.6%)
ACE inhibitors	26 (89.7%)
Calcium-channel blockers	20 (68.9%)
Clopidogrel	17 (58.6%)
ARBs	4 (13.8%)
Other medication	12 (41.4%)

N = 29 patients

*ACE* angiotensin-converting enzyme, *ARB* angiotensin receptor blocker, *CABG* coronary artery bypass surgery, *PCI* percutaneous coronary intervention

**Table 2:** Patient and vessel characteristics according to coronary CTA, FFR_CTA_, FFR_SS_ and FFR

Characteristics	n (%)
Patients with coronary CTA maximum stenosis >50%	23 (79.3)
Patients with minimum FFR ≤0.8	16 (55.2)
Patients with minimum FFR_CTA_ ≤0.8	20 (68.9)
Patients with minimum FFR_SS_ ≤0.8	22 (75.9)
Vessels with coronary CTA stenosis >50%	27 (75.0)
Vessels with FFR ≤0.8	17 (47.2)
Vessels with FFR_CTA_ ≤0.8	22 (61.1)
Vessels with FFR_SS_ ≤0.8	24 (66.7)

N = 29 patients and 36 vessels

*FFR* fractional flow reserve, *FFR*_*CTA*_ fractional flow reserve calculated with the pulsatile flow simulation basing coronary computed tomography angiography datasets, *FFR*_*SS*_ fractional flow reserve calculated with the steady state method

### Analysis of the correlation of FFR_CTA_ with FFR and FFR_SS_

**Fig. 3:**
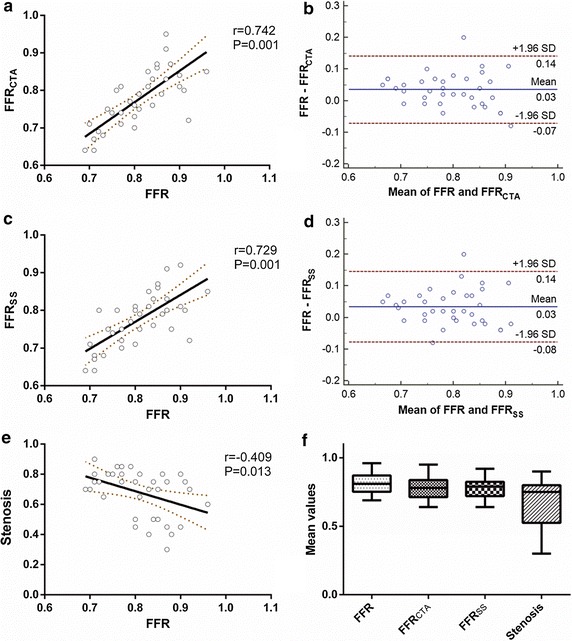
Comparison among CCTA stenosis, FFR_CTA_, FFR_SS_ and invasive FFR on a per-vessel basis. **a** Pearson correlation between FFR_CTA_ and invasive FFR, r was 0.742 with significant difference (*P* = 0.001). **b** Bland–Altman plots of FFR_CTA_ and invasive FFR, mean difference 0.03, standard deviation 0.05. **c** Pearson correlation between FFR_SS_ and invasive FFR, r was 0.729 with significant difference (*P* = 0.001). **d** Bland–Altman plots of FFR_SS_ and invasive FFR, mean difference 0.03, standard deviation 0.06. **e** Pearson correlation between stenosis and invasive FFR, r was −0.409 with significant difference (*P* = 0.013). **f** Mean vlaue of FFR, FFR_CTA_, FFR_SS_ and stenosis

As shown in Fig. 3a, good agreement was observed between the simplified FFR_CTA_ and invasive FFR with a significant difference (r = 0.742, P < 0.001). Furthermore, the Bland–Altman plot presented a slight systematic underestimation of FFR_CTA_ (mean difference 0.03, standard deviation 0.05, P = 0.001, Fig. 3b). A negative correlation was also observed between stenosis and invasive FFR (r = −0.409, P = 0.013, Fig. 3c). However, a similar correlation of the FFR_SS_ obtained from the steady state method with the invasive FFR was found (r = 0.729, P < 0.001), and the Bland–Altman test showed that underestimation was also found in the FFR_SS_ obtained by the steady state method (mean difference 0.03, standard deviation 0.06, P = 0.001). Additionally, the computational efficiency test showed that the computational time spans for the steady state method and simplified pulsatile simulation were 1.2 ± 0.6 h and 2.3 ± 1.2 h, respectively.

### Diagnostic performance of FFR_CTA_, FFR_SS_ and CCTA stenosis for diagnosis of ischaemia

**Fig. 4:**
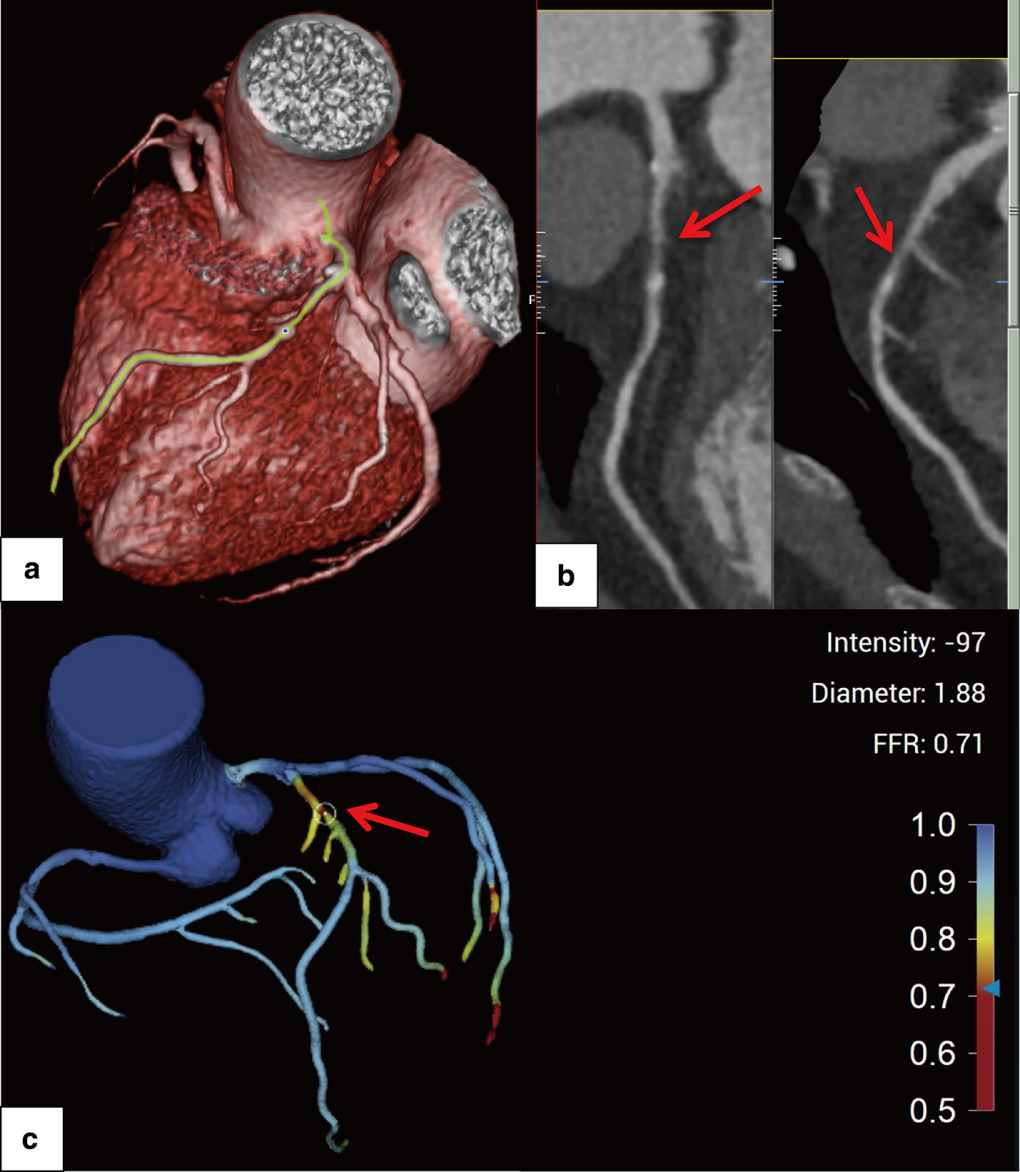
Volume-rendered image (**a**) and multiplanar reformat (**b**) of CCTA and FFR_CTA_ (**c**) of the left anterior descending artery (LAD). CCTA demonstrates stenosis (80% lumen reduction) of the proximal-portion of LAD (*red arrow*) and an FFR_CTA_ value of 0.71. ICA demonstrates a measured FFR value of 0.77

**Fig. 5:**
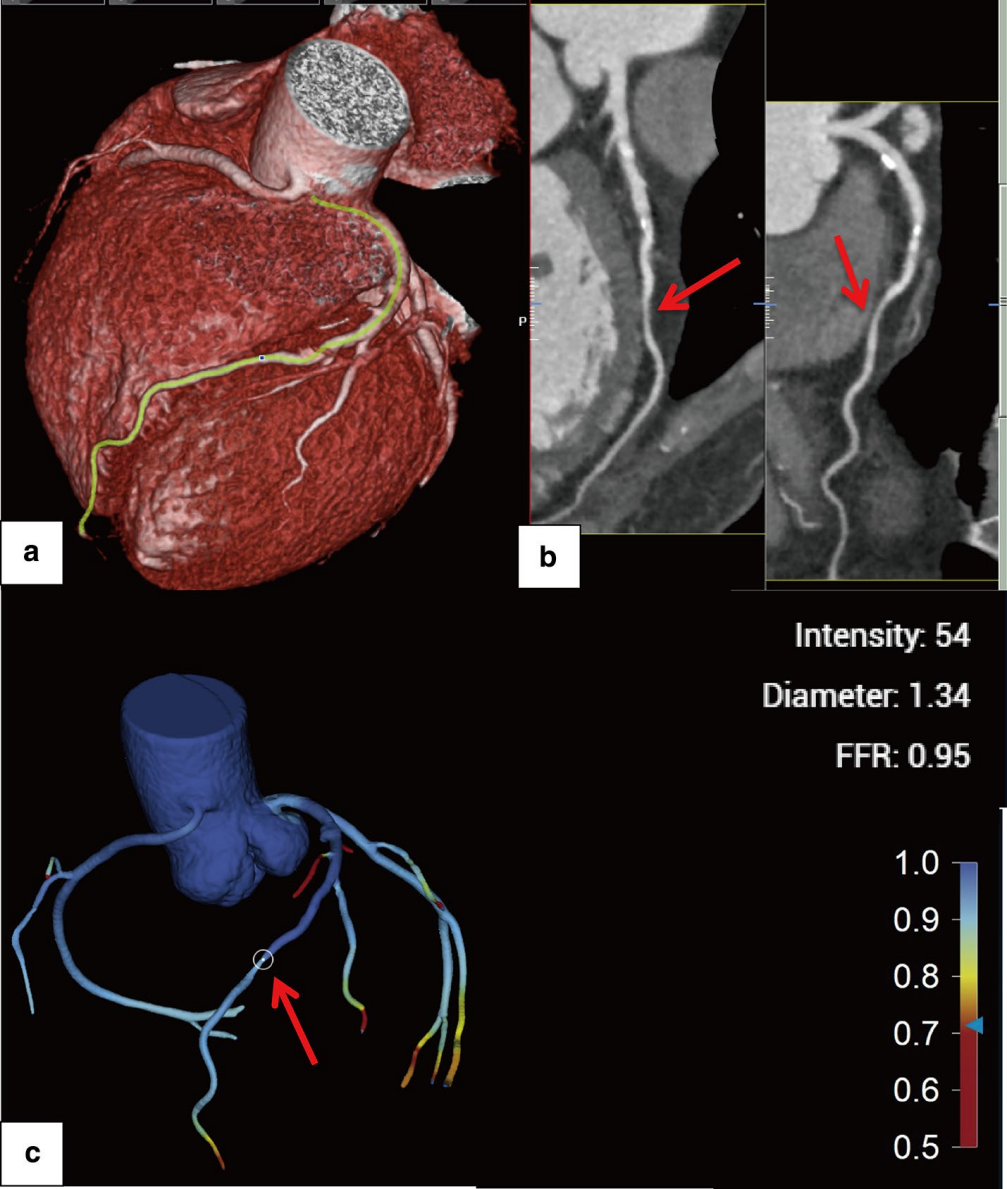
Volume-rendered image (**a**) and multiplanar reformat (**b**) of CCTA and FFR_CTA_ (**c**) of the left anterior descending artery (LAD). CCTA demonstrates stenosis (75% lumen reduction) of the mid-portion of LAD (*red arrow*) and an FFR_CTA_ value of 0.95. ICA demonstrates a measured FFR value of 0.87

**Fig. 6:**
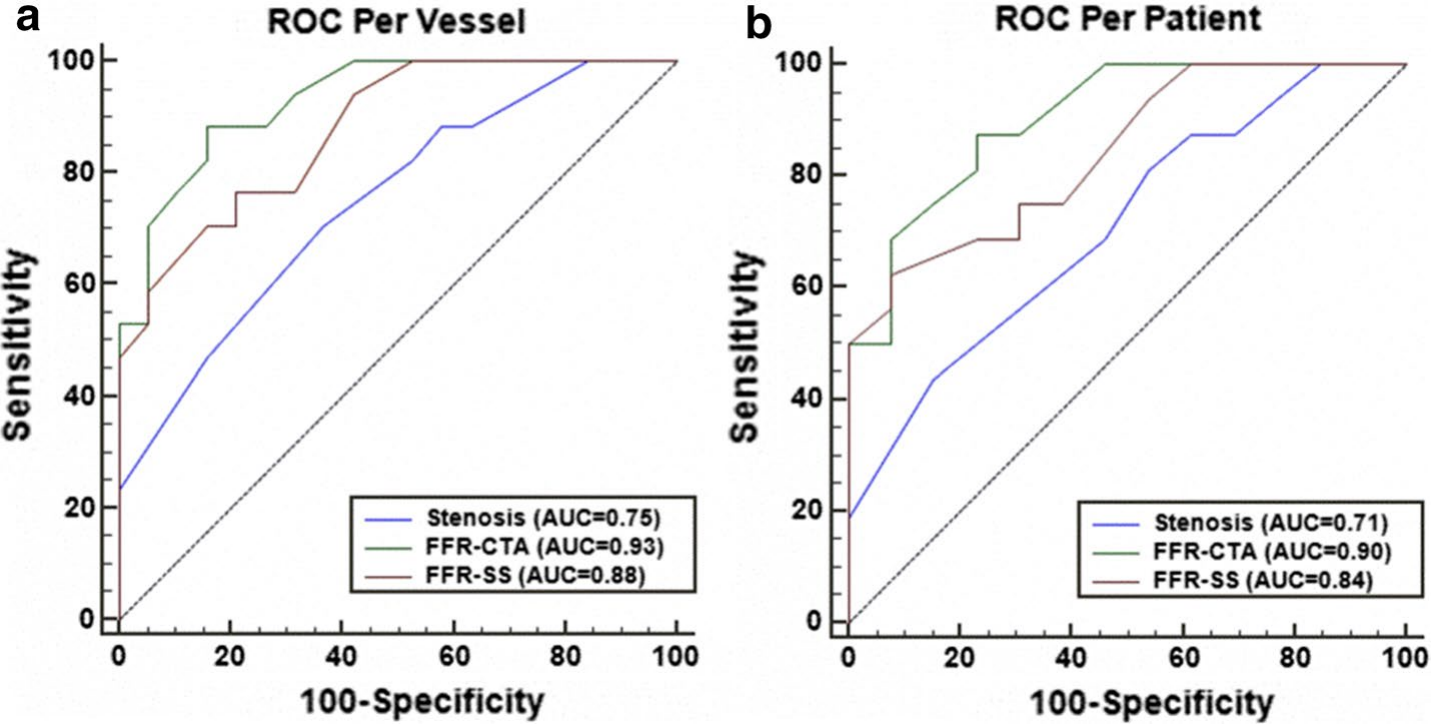
Area under the receiver-operating characteristic curve (AUC) of FFR_CTA_, FFR_SS_ and CCTA stenosis for discriminating ischemia on **a** a per-vessel and **b** per-patient basis separately

The FFR_CTA_ obtained for coronary vessels resulted in 16 true positives (44.4%), 13 true negatives (36.1%), 6 false positives (16.7%), and 1 false negative (2.8%). On a per-patient basis, FFR_CTA_ led to 15 true positives (51.7%), 8 true negatives (27.6%), 5 false positives (17.2%), and 1 false negative (3.4%). The diagnostic performances of FFR_CTA_, FFR_SS_ and CCTA stenosis on a per-patient and per-vessel basis are listed in Table 3. Figures 4 and 5 show representative examples of anatomically obstructive CCTA stenosis with and without ischaemia. In Fig. 6, a higher AUC was observed for FFR_CTA_ and FFR_SS_ compared with CCTA stenosis on a per-vessel basis (0.93/0.88/0.75), as well as on a per-patient basis (0.90/0.84/0.71).

**Table 3:** Diagnostic performance of FFR_CTA_, FFR_SS_ and CCTA on a per-vessel and -patient basis

Measure	Per-vessel			Per-patient		
	FFR_CTA_ ≤0.8 (95% CI)	FFR_SS_ ≤0.8 (95% CI)	CCTA stenosis >50% (95% CI)	FFR_CTA_ ≤0.8 (95% CI)	FFR_SS_ ≤0.8 (95% CI)	CCTA stenosis >50% (95% CI)
Accuracy	80.6 (64.9–90.2)	75.0 (58.9–86.3)	61.6 (44.8–75.2)	79.3 (61.6–90.1)	72.4 (54.3–85.3)	62.1 (44.0–77.3)
Sensitivity	94.1 (71.3–99.9)	94.1 (73.0–98.9)	88.2 (63.6–98.5)	93.7 (69.8–99.8)	93.7 (71.7–98.9)	87.5 (61.7–98.4)
Specificity	68.4 (43.4–87.4)	57.9 (36.3–76.9)	36.8 (16.3–61.6)	61.5 (31.6–86.1)	46.2 (23.2–70.9)	30.7 (9.1–61.4)
PPV	72.7 (49.8–89.3)	66.7 (46.7–82.1)	55.6 (35.3–74.5)	75.0 (50.9–91.3)	68.2 (47.3–83.6)	60.9 (38.5–80.3)
NPV	92.9 (66.1–99.8)	91.7 (64.6–98.5)	77.8 (40–97.2)	88.9 (51.8–99.7)	85.7 (48.7–97.4)	66.7 (22.3–95.7)
+LR	2.9 (1.5–5.8)	2.2 (1.7–2.9)	1.4 (1.0–2.1)	2.4 (1.2–4.9)	1.7 (1.3–2.3)	1.3 (0.8–1.9)
−LR	0.09 (0.01–0.6)	0.1 (0.0–0.8)	0.32 (0.08–1.3)	0.1 (0.01–0.7)	0.1 (0.0–1.4)	0.4 (0.09–1.9)

*CI* confidence interval, *+LR* positive likelihood ratio, −*LR* negative likelihood ratio, *NPV* negative predictive value, *PPV* positive predictive value

## Discussion

At present, the fractional flow reserve (FFR) has been regarded as the gold standard in the assessment of haemodynamic characteristics for coronary stenosis [5], and it is recommended for making clinical treatment decisions before coronary revascularization [6]. FFR is applied to guide management in less than 10% of PCI cases, especially in developing countries, because of the high cost and invasive procedure [8]. In recent years, with the development of computational fluid dynamics (CFD), it is possible to calculate the coronary flow and pressure from anatomic imaging data [14]. FFR_CTA_ has emerged as a new non-invasive method and has been investigated worldwide.

In this study, FFR_CTA_ was calculated by implementing pulsatile flow simulation with novel parameter estimation. By comparing the correlation of FFR_CTA_ and FFR_SS_ with the invasive FFR in patient-specific coronary arteries, the accuracy of the FFR_CTA_ calculated by using pulsatile flow simulation in the present study is slightly higher than that using steady state simulations [19]. The area under the curve (AUC) was used to evaluate the diagnostic performance per-vessel and per-patient. Our result showed that FFR_CTA_ has higher diagnostic performance and a larger AUC than CCTA stenosis alone; the result was equivalent to Norgaard and Zhang’s research except for a decreased specificity. However, the overall diagnostic performance in the present study was lower compared to previous studies [18, 19]; this may mainly be related to the calcification of the stenosis in the present study that is commonly found in stenosis in the clinic. The artefacts from calcification decrease the apparent lumen and lead to narrow coronary segmentations, resulting in lower FFR_CTA_ values and an increased proportion of false positive cases [29]. Overall, the results presented in our study showed that it is possible to obtain FFR_CTA_ based on the simplified method with pulsatile flow simulation and a novel boundary condition within a reduced computational time.

The calculation of FFR derived from CT imaging data represents an alternative approach in the assessment of haemodynamic characteristics for coronary stenosis. In clinical practice, the severity of stenosis shows a poor relationship with ischaemia [30], as shown in Fig. 3e. Especially in patients with stenosis in the intermediate range (30–70%), it is hard to judge whether the severity of the stenosis would lead to ischaemia without an invasive FFR measurement through an expensive coronary pressure wire. The application of FFR_CTA_ is conducive to reducing the false-positive cases caused by coronary CT angiography findings and cutting down the need for a second diagnostic examination. Despite an incremental diagnostic performance of FFR_CTA_ having been reported by several clinical trials, its application is still limited because of the demanding process. Comparing the pulsatile flow simulation that was used in the DISCOVER-FLOW [15], DeFACTO [16] and NXT [17] trials, the simplified method applied in the present study can reduce the simulation time span significantly and maintain a superior diagnostic discrimination characteristic. However, the value of the simplified method was debatable in that the transient fluid dynamic analysis is still an effective tool, especially for the complex distribution of multiple stenoses. In addition, the high quality of the CT image and the consistent CTA protocol are also important to improved diagnostic performance of FFR_CTA_ [17]. With the use of an MDCT volumetric scanner with 320-detector rows, finer detector elements of 0.5 mm compared with many other CT scanners can be achieved in this study.

In clinical practice, several tests have been established as non-invasive methods to provide functional diagnostic information, such as single photon emission computed tomography (SPECT), coronary magnetic resonance imaging (cMRI) or stress echocardiography. These methods can provide useful information on patient prognosis, and thus they have been recommended for evaluating patients with symptoms in the guidelines [31]. Several studies have investigated the ability of these tests to identify ischaemia. In Jogiya’s research, the sensitivity, specificity, and diagnostic accuracy of cMRI for the detection of significant CAD were 91, 90, and 91%, respectively [32]. In another study of early dipyridamole stress, for myocardial SPECT to detect residual stenosis, the sensitivity and specificity of SPECT to detect the functionally and morphologically significant residual stenosis were 92 and 31% and were 83 and 29%, respectively [33]. In Jung’s study of dobutamine stress echocardiography, a sensitivity of 48% and a specificity of 73% were reported [34, 35]. However, these methods do not visualize the stenotic coronary arteries and cannot provide haemodynamic information of the individual coronary lesions compared with CCTA and FFR_CTA_. With continually rising healthcare costs, more attention is focused on the cost effectiveness of procedures. The present study supported that, as an alternate diagnostic parameter, the FFR_CTA_ calculated by this simplified method has the potential to be an available gatekeeper to ICA and revascularization compared with the invasive FFR measurement and could reduce healthcare costs for patients suspected of having CAD at the same time. In addition, FFR_CTA_ can also be used to predict the haemodynamic changes resulting from percutaneous coronary intervention or coronary artery bypass graft. Likewise, the method established in our study has the potential to be generalized to peripheral vascular disease, such as carotid, renal and cerebral vascular stenosis.

Several limitations exist in the present study. There are several outliers observed in Fig. 3a, b. The reasons may lie in the following aspects: (1) the limited resolution in the small vessel of the CCTA could result in the deviation between reconstructed geometries and actual anatomy, contributing bias to the calculations; (2) the timespan for the pulsatile flow simulation was longer than that for the steady state simulations, so a more efficient algorithm is required to advance the clinical implementation of FFR_CTA_; (3) because the coronary arteries are fixed on the heart wall, the deformation of the vessel walls caused in the end-diastolic phase is not included; (4) the small size of samples obtained in the present study prevented us from further analyzing the stenosis in the intermediate range (30–70%), which showed the poorest relationship with ischaemia in the clinic; and (5) because patients with acute coronary syndromes or previous coronary intervention or bypass surgery were not included in the present study, whether this method can be applied to these patients still needs to be studied.

## Conclusion

In this study, a simplified method algorithm was employed to calculate FFR_CTA_; we observed good correlation and an acceptable mean difference between FFR_CTA_ and invasive FFR, as well as a better diagnostic performance of FFR_CTA_ in diagnosing ischaemia-causing stenosis in the clinic. By implementing this new boundary condition, the simplified FFR_CTA_ calculated with pulsatile flow has the potential to be an alternate and accurate diagnostic parameter in the assessment of the haemodynamic characteristics for coronary stenosis.

## Abbreviations

CAD: coronary artery disease
ICA: invasive coronary angiography
FFR: fractional flow reserve
FAME: fractional flow reserve versus angiography for multivessel evaluation
PCI: percutaneous coronary intervention
CCTA: coronary computed tomographic angiography
CFD: computational fluid dynamics
LPM: lumped parameter model
MUMPS: multifrontal massively parallel sparse direct solver
PPV: positive predictive value
NPV: negative predictive value
+LR: positive likelihood ratio
−LR: negative likelihood ratio
AUC: area under the receiver-operator characteristics curve
SPECT: single photon emission computed tomography
cMRI: coronary magnetic resonance imaging
